# Exploring the Impact of Virtual Reflection Groups on Advanced Practice Nurse Students During the COVID-19 Pandemic: Focus Group Study With Master’s Students

**DOI:** 10.2196/40418

**Published:** 2022-09-15

**Authors:** Jofrid Berit Høybakk, Andréa Aparecida Gonçalves Nes, Monica Evelyn Kvande, Marianne Trygg Solberg

**Affiliations:** 1 Lovisenberg Diaconal University College Oslo Norway

**Keywords:** advanced practice nurse, competence, COVID-19, professional discussions, qualitative study, virtual reflection groups, interviews, learning, development

## Abstract

**Background:**

In the master’s program of advanced practice nursing at a Norwegian university college, the learning activity reflection groups were converted into virtual reflection group (VRG) meetings during the COVID-19 pandemic. Regardless of the students’ clinical practices in different hospitals, they could participate in the same VRG meeting on the web together with the educator from the university college, and the clinical supervisors were invited to participate. The students were in the process of developing the core competence required in their role as advanced practice nurses (APNs), and they had increased responsibility in the implementation of the VRG meetings.

**Objective:**

In this study, we aimed to explore how master’s students of advanced practice nursing experienced VRG meetings during the COVID-19 pandemic.

**Methods:**

A qualitative exploratory design was adopted using focus group interviews. A group of students in the master’s program of advanced practice nursing participated in an interview that lasted for 60 minutes. They had experienced participating in the VRG meetings following a rigorous guide during their clinical practice. The data from the focus group were analyzed using qualitative content analysis.

**Results:**

The main findings of this study highlighted the importance of structure in VRG meetings, the role of increased responsibility in students’ learning processes, the development of APN students’ competencies, and increased professional collaboration with clinical supervisors. The APN students and clinical supervisors also continued their discussions in the clinical setting afterward, which strengthened the collaboration between students’ education in the master’s program and their clinical practice.

**Conclusions:**

VRG meetings gave the students the opportunity to lead professional discussions while reflecting thoroughly on the chosen patient cases from clinical practice. They experienced receiving feedback from fellow students, supervisors, and educators as stimulating their critical thinking development.

## Introduction

### Background

Recently, the world has experienced a pandemic of a new disease, COVID-19, and health professionals, especially nurses, have had to face challenges they had not experienced before [1,2]. The COVID-19 pandemic has brought about radical changes to education systems worldwide. During this period (2020-2022), a rapid increase in distance learning has taken place, in which technology-supported pedagogical methods have been developed, along with the use of digital tools for web-based education and communication between students and educators [2-5]. In a Norwegian master’s program in advanced practice nursing, a new alternative learning activity, the virtual reflection group (VRG), supported by technology, was introduced to students during the COVID-19 pandemic [6]. The VRG learning activity aims to enhance students’ development of the competencies required in their role as advanced practice nurses (APNs). This advanced competence is necessary for the care and management of patients facing complex health care demands [7,8]. The complicated issues affecting patients with one or several chronic diseases or comorbidities, together with a significant increase in the ageing population, pose several challenges in health care [9,10]. An APN is a generalist or specialized nurse who has acquired the expert knowledge base, complex decision-making skills, and clinical competencies for advanced nursing practice with a minimum of a master’s degree [11]. The APN role is in development both internationally and nationally in Norway. Owing to the changing tasks and increased responsibilities expected of nurses in Norwegian hospitals, there is the need for APNs with broad clinical competence at an advanced level. APNs are especially required to care for patients with complex demands, including unresolved or acute clinical problems [12].

The competencies that APN students need to develop during their education include the knowledge to conduct systematic clinical examinations and health assessments, the skills to handle complex patient situations independently, and the ability to assess the severity of the patient’s health, including changes over time [13]. Consequently, APN students must develop a professional and autonomous role by acquiring advanced knowledge in critical thinking, clinical decision-making, and comprehensive assessment [7]. The APN core competence also includes leadership, collaboration, guidance, and coaching, along with evidence-based practice [13].

The Norwegian master’s program in APN is in line with the European requirements of 120 credits [14]. To improve students’ learning process throughout their master’s education, constructive alignment is incorporated at the university college as a pedagogical learning model, assuring logical interaction between learning activities, assessment, and learning outcomes [14,15]. The learning outcomes are developed to help students achieve the core competencies necessary to manage complex patient situations [14]. The master’s program consists of alternating lectures and 3 periods of clinical practicums, from 8 to 11 weeks.

The learning outcomes for students in the clinical practicums were as follows: (1) further develop and act independently based on the knowledge of the role of advanced clinical nurses, (2) justify and argue for one’s own choice of action together with others involved, (3) apply knowledge and skills to implement and independently assess the results of knowledge-based nursing practices, and (4) analyze and critically reflect on their own and others’ actions in acute, serious, or critical situations [14]. The students are gradually developing the necessary competence to assume the role of APN, supported by a clinical supervisor. To facilitate the integration of theory and experience in clinical practice, APN students participate in reflection groups [14].

The reflection groups are a learning activity that supports the collaborative learning of students, together with their educator, and their clinical supervisors are also invited to participate [6]. Through reflection, the students can express their views and arguments, along with their previous experiences, which the group members can either confirm or challenge [16]. The study by Schön [17] distinguished between reflection-in-action and reflection-on-action concepts. Reflection-in-action means that when something unexpected or problematic happens, the person thinks about it and adapts their actions to the current situation, whereas reflection-on-action means thinking back to what happened in the situation and why it occurred to better understand it. The study by Edwards [18] added 2 more steps to the Schön [17] reflection process: reflection-before-action and reflection-beyond-action. The APN students practice reflection-before-action when they prepare for meeting in the reflection groups. Later, reflection-beyond-action in the reflection groups allows APN students to use an experienced story or case to enhance their self-exploration and awareness, which promotes life-long learning, advances practice development, and leads to transformative learning [18]. Reflection is a particularly important process in developing professional competence in clinical practice [19].

**Figure 1:**
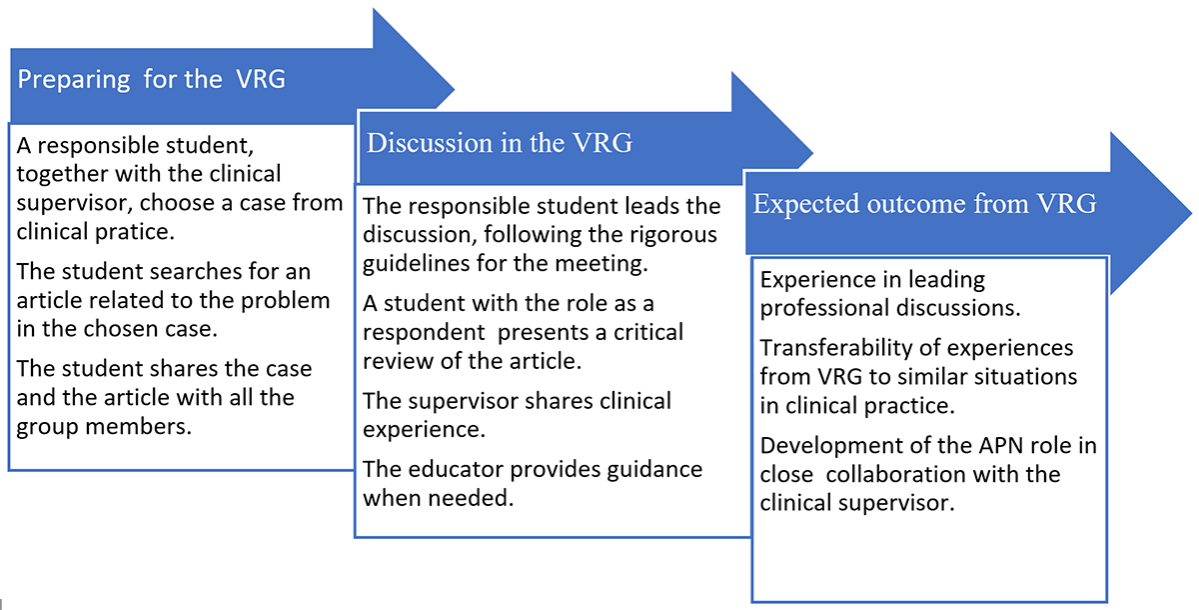
A model illustrating the learning process and responsibilities of students participating in virtual reflection group (VRG) meetings. APN: advanced practice nurse.

Originally, the reflection group meetings in the master’s program were arranged on campus, and therefore, the student’s supervisor could not participate [14]. The new learning design with web-based meetings has recently replaced campus-based reflection groups. The web-based VRG meeting, which uses rigorous guidelines, was tested in a pilot project from 2019 to 2020 for APN students in critical care. In 2021, the VRG was implemented in the master’s program during the COVID-19 pandemic lockdown [6]. The benefit of the VRG meetings was that even if the students’ clinical practice took place in different hospitals, they could participate in the same VRG meeting on the web, together with the educator from the university college, using the digital platform Zoom, and the clinical supervisors could also participate remotely [6]. In these meetings, the students were in the process of developing core competencies for their role as APNs. Their learning process, showing their increased responsibility in VRG meetings, is illustrated in Figure 1.

### Objective

In this study, we aimed to explore how the APN master’s students experienced the VRG meetings during the COVID-19 pandemic. The following were research questions:

- How did the master’s students experience the VRG meetings in the development of their competence as an APN?
- How did the master’s students experience the collaboration with the clinical supervisor in conducting the VRG?

## Methods

### Design

This study used a qualitative exploratory design with focus group interviews. An advantage of using focus group interviews is that they provide the group dynamics and synergies for accessing rich information from the students [20,21]. The interactions between the participants in the focus group can provide insight into a range of students’ opinions, perceptions, and attitudes, which might be less accessible in individual interviews [20]. The group dynamics and interactions were expected to help the master’s students clarify their experiences in participating in VRG meetings during their clinical placement.

### Setting and Participants

This study was conducted at a Norwegian university college with students in a master’s program in advanced practice nursing during the COVID-19 pandemic. All students participated in a VRG using rigorous guidelines [6]. They had completed the third placement of their clinical practice in different hospitals and units in East Norway, and they were invited to participate in the study after receiving both oral and written information. A total of 6 female students gave their informed consent to participate in the focus group interview.

### Data Collection

The focus group interview was conducted in October 2021, immediately after the students had completed their clinical practice, which included participating in the VRG. The focus group interview was performed on-site in a meeting room at the university college, with the participants seated around a table to indicate the equal importance of each participant’s contributions. Neither the moderator (MTS) nor the comoderator (MEK) was responsible for educating the master’s students, which encouraged an honest and open dialogue during the interview. The open-ended questions of the interview guide (Textbox 1) were developed to answer the research questions.

During the interview, the participants were encouraged to ask questions, exchange anecdotes, and comment on each other’s experiences and views, and the group dynamics helped the participants to create narratives [20,21]. The interview was audio-recorded and lasted for 60 minutes, which is a common duration of a focus group interview [20].

**The interview guide.**

**Main questions:**

Can you talk about your experiences participating in the virtual reflection group (VRG) meetings?

- What are the benefits and limitations of the VRG meetings?
- What was your experience of following a guide for conducting the VRG meetings?
- What learning outcomes did you achieve from the VRG meetings regarding the development of your role as an advanced practice nurse (APN)?
- How did the professional discussion contribute to your development as an APN?

**Supporting questions:**

Different roles are included in the implementation of a VRG: What expectations did you have in advance regarding

- leading the professional discussion when conducting the VRG?
- including the clinical supervisors in the discussion to share their experiences?
- your role as respondent?

### Ethical Considerations

The study was approved by the faculty at the university college and registered at the Norwegian Centre for Research Data (reference number NSD 578229). All participants provided informed consent to participate in the focus group interviews, and they were assured of full confidentiality and anonymity [21]. The moderator (MTS) and comoderator (MEK) ensured that no names or other personal information remained in the audiotape.

### Data Analysis

The data from the focus group interviews were transcribed verbatim by the first author (JBH) as a starting point for further analysis. All authors took part in the process of data analyses and read transcript data several times to gain insight into the content. The data were analyzed inspired by Graneheim and Lundman [22], using qualitative content analysis.

In the first step, the data were divided into meaning units related to the purpose of the project. The identified meaning units were then condensed into descriptions close to the text. In the second step, the underlying meanings of the condensed meaning units were interpreted, and all authors agreed upon the division of the results into subthemes and themes. During the analysis, we moved forward and backward among the meaning units, subthemes, and themes [22,23]. An example of the step-by-step analysis used to develop the theme of the *VRG meeting structure* is presented in Table 1.

**Table 1:** Example illustrating the analysis process from meaning unit to theme.

Meaning unit	Condensed meaning unit description close to the text	Interpretation of the underlying meaning	Subtheme	Theme
Participating in the VRG^a^ meeting by presenting the case and leading the meeting	Following the rigorous guidelines provided structure when conducting the VRG meetings	Using rigorous meeting guidelines contributed to the distribution of the disponible time and clarified the roles for participants	Following a rigorous guideline for the VRG meeting	VRG meeting structure
Digital communication in groups may inhibit the spontaneous, unstructured discussion via Zoom, as everyone who participated had to wait for their turn to speak	Challenge leading a web-based discussion and being responsible for all the participants in the discussion	Following the guideline ensured that all group members were included in the discussion. Waiting for one’s turn to participate in the discussion gave time for reflection.	Following a rigorous guideline for the VRG meeting	VRG meeting structure
Useful to follow the guideline, got more responsibility for the implementation of the reflection groups.	The guidelines gave structure when leading the discussion in the meeting.	The student was responsible for leading the professional discussion, with reflection on the experienced case.	Delegation of responsibility to the VRG members	VRG meeting structure
In the VRG, we went through both the article and the patient case. We also started a discussion about the case.	It was instructive both to present the patient case and to be the respondent.	Both the roles of leading the discussion and being a respondent increased the student’s responsibility when conducting the VRG.	Delegation of responsibility to the VRG members	VRG meeting structure

^a^VRG: virtual reflection group.

### Trustworthiness

All authors have extensive research experience. The second author (AAGN) worked in an undergraduate nursing program, whereas all the other authors worked in a postgraduate critical care nursing education program. The first author (JBH) is in an assistant professor program, and the others are associate professors. The moderator and comoderator, in line with Polit and Beck [21], limited their reactions to the participants and tried to avoid influencing the answers, both verbally and nonverbally. The participants talked at length about their experiences and were not afraid to express their diverse perceptions. The comoderators took notes during the interviews to supplement the verbal transcripts in the analysis discussions. The different levels of expertise deepened the transparency of the results in this study, as all the authors participated in the analysis and agreed on the results.

## Results

### Overview

**Figure 2:**
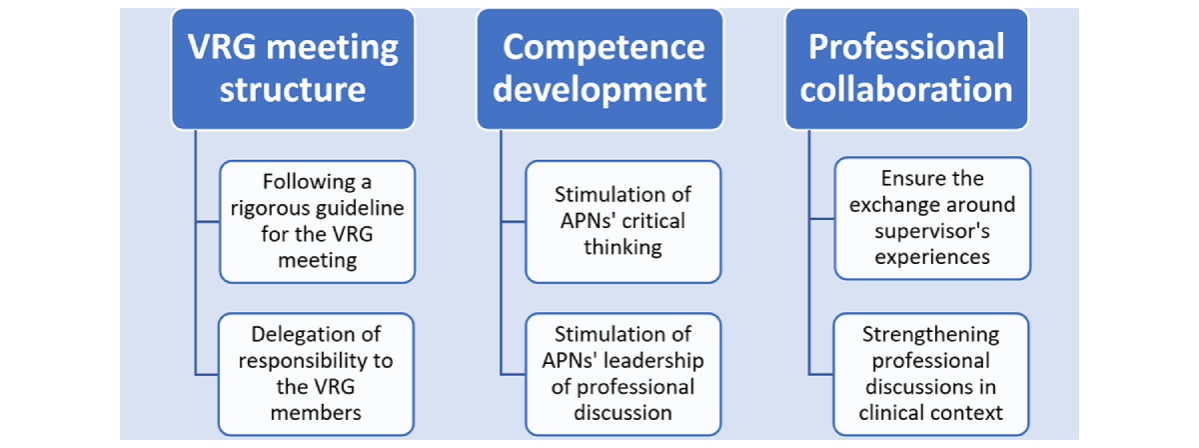
Results with themes and subthemes. APN: advanced practice nurse; VRG: virtual reflection group.

This study explored how APN master’s students experienced participating in a VRG meeting during the COVID-19 pandemic. We identified 3 themes and 6 subthemes in the data, as illustrated in Figure 2. To ensure anonymity when presenting the results, references to individual participants’ statements use nonidentifying letters (A-F), representing the individual APN student.

### VRG Meeting Structure

#### Preparing for the VRG Meeting

The responsible student experienced the importance of having enough advance time to prepare the selected patient case together with the clinical supervisor and to look for a research article that could highlight the chosen patient situation. The flexibility to choose the time for the VRG meeting was also important to facilitate and ensure supervisor participation. Furthermore, the responsible student described the case with anonymized information and saved it on a secure digital learning platform (Canvas). The group members accessed the patient case in advance in preparation for the VRG meetings.

#### Following a Rigorous Guideline for the VRG Meeting

In the data, we found that all the participants became familiar with the new structure to follow during the VRG, as outlined in the meeting guideline. They found it useful to follow the rigorous meeting guideline in terms of providing structure when conducting the VRG meetings using web-based digital communication. The students felt that the guideline reinforced their responsibility to be prepared for their different roles, including the responsible student, the respondent, and the remaining students in the VRG, together with the clinical supervisor and educator. One of the participants compared the experiences of the regular reflection group and the VRG meeting, stating the following:

I achieved more learning outcomes now, participating in VRG meetings by presenting the case, leading the meetings and also, as a respondent, giving feedback on the research article related to the patient case. I think I gained many more benefits from this type of reflection group.

Participant C

It appears that using a rigorous meeting guideline contributed to the distribution of disponible time and clarification of the participants’ roles.

The students perceived both benefits and challenges of VRG meetings. One student stated the following:

Digital communication in groups may inhibit the spontaneous, unstructured discussion via Zoom, as everyone who participated had to wait for their turn to speak, in contrast to being in the same physical room.

Participant C

However, there were also descriptions related to the benefits of waiting for one’s turn to speak: “What you say may be more thoughtful, but you may also censor yourself because you have thought too much about it” (Participant D).

Furthermore, as the reflection groups took place digitally, some students missed meeting each other face to face, which provides opportunities for spontaneous discussion. In addition, the experience of safety in the group was perceived as important. The confidence in each other was generated by previous contact with group members. Following the guideline ensured that all group members were included in the discussion.

#### Delegation of Responsibility to the VRG Members

The participants experienced as positive the distribution of different roles and responsibilities for the VRG members. In every meeting, a different student was responsible for presenting the chosen case and leading the discussion, whereas another student was the respondent, providing critical feedback on the chosen article. The remaining students participated in the discussion together with the clinical supervisor and the educator. One of the students elaborated on the roles:

In our reflection group, we went through both the article and the patient case. Further, we started a discussion about the case, which was the reflection itself. I expected responses on the case and the chosen article I had presented.

Participant B

The responsible student gained experience in finding and choosing relevant research articles that highlighted a patient’s case. The student in the respondent’s role was learning to be critical of research. Both students learned to evaluate and reflect on the research articles in addition to knowing how and where to apply the acquired knowledge in an educational and clinical context.

One of the students in the respondent role reported about focusing on the chosen research article:

The respondent role was limited to giving feedback on the relevance of the selected research article to the chosen patient case and its methodological approach, and not so much focus was given to the content of the chosen patient situation.

Participant D

Another student reported that the achievement of the learning outcomes was possible because of the delegated responsibilities regarding leading the meeting and being a respondent:

Both roles increased (the student) responsibility when conducting the VRG, and it was instructive both to present the patient case and to be the respondent.

Participant A

When leading the discussion, students experienced that the guideline provided a structure for the meeting and helped a great deal with the distribution of the tasks. The students experienced that the meeting guidelines provided a clear structure for the distribution of time and responsibilities when conducting the VRG meeting. The student responsible for presenting the case was also responsible for leading the professional discussion through reflection, which occurred after the respondent’s feedback on the chosen research article for the studied patient case.

### Competence Development

#### Preparing for Leading the VRG Meeting

Preparing in advance for the VRG meetings, together with the clinical supervisor, stimulated the students’ reflection, especially when describing the patient situation. During the VRG, the students acquired experience in leading academic discussions and were challenged in their development of critical thinking.

#### Stimulation of APNs’ Critical Thinking

The students experienced an increased focus on the APN role in the VRG meetings. They were in the last of the 3 periods of clinical practice and had achieved advanced knowledge and developed the competence to make complex clinical decisions and use critical thinking at a higher level. They reported the development of academic argumentation by participating in the VRG meetings:

I like to argue, which becomes much easier when you can use research to justify the issues. For example, when it comes to change in routines...

Participant B

In the data, we found that the students were more self-confident with the competence they acquired through their master’s education, which was an ongoing process. They found that the VRG meetings were useful as they were challenged to argue and express their opinions on different issues concerning clinical patients, which stimulated their development of critical thinking. The flexibility and safe environment of the VRG meetings were also underlined. They could join in a discussion based on their clinical practice, and they could stay in it even if their arguments were flawed. In addition, they felt more confident in their competence regarding ethical issues with reflection on ethical dilemmas from clinical practice.

The students reported how the content of the VRG meetings, which were based on actual patient cases, relevant research articles, and feedback from their peers, stimulated their reflection skills and increased their knowledge:

To elaborate a patient case and find a relevant article that you will receive feedback on stimulates reflection: Is the case I have written good enough? Is the research I have found good enough? It is an advantage to get a “second opinion” from another APN who looks at the content with different eyes than I do.

Participant C

The students reflected together, thus contributing to their different opinions, whereas one of the students gained experience in leading the discussions.

#### Stimulation of APNs’ Leadership of Professional Discussion

The students were responsible for leading the VRG meetings, and they facilitated an in-depth discussion of the presented patient case. They acquired experience in leading professional discussions according to the meeting guidelines. They also reported positive and instructive experiences from receiving feedback on the article and leading the discussion focused on the chosen patient situation.

The data show that some of the participants were afraid and insecure in leading the VRG meeting, as they were responsible for presenting the case to their fellow students, clinical supervisors, and educators. A student (Participant F) reported that it was useful to be a little insecure while practicing the leading role in front of the other group members. Another student reflected on the uncertainty they experienced leading the professional discussion in the VRG meetings:

The first time, I was unsure about my leading role in relation to the educator role, about how strong a position I should take in leading the discussion.

Participant D

Another student experienced that it was a challenge to lead the virtual discussion with fellow students because of the need to pay attention to the task at hand and, at the same time, ensure that everyone had the chance to say something in the discussion. They perceived that the size of the VRG was important to ensure the achievement of learning outcomes, as 1 student commented:

I think practising in a small group is good, compared to a large group, which can be scary. Small groups, where you are confident in each other, are fine.

Participant A

They found that the sensation of safety related to small groups increased the activity and gave all members an opportunity to speak. One student (Participant B) expressed that she dared to say more in a small group and with people that everyone knew.

Participating in VRG meetings gave the students the experience of leading professional discussions. One student (Participant F) mentioned that these experiences were useful in developing their competence as future APNs. In the VRG meeting discussions, they experienced keeping their focus on the APN role and responsibilities in close collaboration with their clinical supervisor.

### Professional Collaboration

#### Ensure the Exchange Around Supervisor’s Experiences

The clinical supervisor participated in the VRGs and shared experiences that challenged the students to think critically about how to apply research in clinical practice. The VRG meeting structure made the participation of the clinical supervisor possible to the benefit of the responsible student and the other students. The students gave positive feedback regarding their experiences in collaboration with the clinical supervisor in terms of preparing for VRG meetings and leading professional discussions. The students perceived it as very useful to get feedback directly from the supervisors, who could contribute their clinical experience and identify the relevance of the chosen research article to the selected patient case. One student elaborated on the benefit of having the clinical supervisor as a participant in the VRG meeting, stating the following:

It helps to get a different view of the case. The supervisor can participate and give input on how it works in everyday life, justifying why it works and why they do things the way they do.

Participant F

Another student chose a case in collaboration with the clinical supervisor, in which the topic was pain, and she elaborated on the learning outcomes:

The more I read, the more exciting it became. It was interesting to get feedback from the respondent on the case. I heard several points of view on how we could choose to solve the situation. To discuss around a case was very instructive, and several thoughts came up. It was an exciting case.

Participant C

When selecting cases from clinical practice, the students had to select a research article that could clarify the chosen case. They experienced excellent collaboration with their clinical supervisors, who helped them find a relevant research article for the chosen patient case. One student experienced it in the following manner:

The clinical supervisor came up with unique points of view from the unit the case was drawn from and could explain the background, why it had turned out the way it had, and what choices were made that the rest of us could only comment on; it was absolutely valuable.

Participant D

The students felt that the clinical supervisor could bring out other points of view on the case, as seen from the practice side, and elaborate on and justify the assessment and management that were done based on an APN’s role and responsibilities.

The students experienced that the supervisor’s input was valuable to the choice of the research article. This input was based on a critical assessment of the research article that evaluated its relevance to the chosen patient case. One of the students (Participant D) stated, “There was a good collaboration with the practice supervisor who helped me find a relevant research article that could highlight the chosen patient case.”

Another student (participant B) stated, “To get feedback from the supervisor on research related to the patient case, for example, if it was a good article or if I should choose something else.” The students felt that they gained relevant competence to develop as an APN from their experiences of participating in the VRG meetings regarding linking research to clinical situations.

#### Strengthening of Professional Discussions in Clinical Context

According to 1 student (Participant E), the topics that were raised in the VRG meetings had ripple effects on clinical practice, as the supervisors brought the topic back to their units. One student (Participant F) illustrated these effects by referring to the topics raised by the supervisors about their clinical practice that were discussed in the VRG meetings. The supervisors from clinical practice who participated in the VRG meetings also showed commitment, as 1 student elaborated on as follows:

The supervisor later discussed in clinical practice the topic that had been chosen (for the VRG) and afterwards how the situation had been followed up.

Participant A

Another student reflected on the compliance between the possible procedure discussed for the patient case based on the research article and the procedure performed by the supervisor in clinical practice:

My supervisor found the topics I raised in the VRG interesting. I located a research article with a few points that interested the clinical supervisor, and the supervisor brought this knowledge back to the clinical practice.

Participant D

In the VRG meetings, APN students, clinical supervisors, and educators had the experience of reflecting on clinical practice situations. They linked relevant research-based knowledge to current patient cases from the unit. The discussions in the VRG meetings positively influenced decision-making related to patient cases in clinical practice, which strengthened the professional discussion related to the clinical context.

## Discussion

### Principal Findings

This study aimed to explore how APN master’s students experienced VRG meetings, which were implemented for all master’s students during the COVID-19 lockdown. By participating in the VRG meetings and experiencing different roles, the APN students increased their responsibility, both by leading and taking part in professional discussions about issues derived from clinical practice. The APN students experienced rigorous guidelines as useful for organizing meetings with a structure that facilitated the distribution of time [6]. The students took turns assuming responsibility for leading the meeting discussions, which they reported as a useful experience in preparing for their role as APNs. As described in the literature, leading professional discussions is one of the core competencies required in the APN role, and the meetings allowed students to develop advanced abilities in leadership and collaboration [13], a skill that was identified in a previous study to be highly important in APN education [7]. The students reported that they experienced increased learning outcomes by participating in VRG meetings and collaborating in various roles.

By changing from on-site meetings at the campus to distance learning through web-based meetings that followed rigorous guidelines in conducting the VRGs, the students took on increasing responsibility, which improved their learning process and learning outcomes in the clinical practicum. These findings are in line with the results of another study that explored how students adapted to the changes associated with the pedagogical transition to distance learning and use of digital tools because of the COVID-19 pandemic lockdown [3]. The students were trained in digital communication, distance learning, and the use of digital tools during the lockdown [1,2,4,5]. Our APN students also experienced the COVID-19 pandemic by reducing the face-to-face physical contact between them in different clinical placements and the contact between the students and educators, results that are in line with other studies [2,5]. In addition, the students participating in the VRGs experienced that it could be challenging to discuss issues in a digital setting rather than face to face, as spontaneous responses in the discussions were less likely [2]. Despite the challenges in web-based communication, the students pointed out some advantages. In the VRG meetings, the students followed rigorous guidelines, including waiting for their turn to speak while others were talking. By waiting for their turn, the students experienced extra time to reflect on the issues they were discussing, which they experienced as a benefit.

The results of this study show that by participating in VRG meetings, the APN students were challenged to reflect on the competence they had developed in their master’s education. They also experienced increased awareness of the APN’s role in complex patient situations during clinical placement. Preparing the case in advance of the VRG meeting contributed to the students’ in-depth knowledge, as they used the content from a research article for professional argumentation in discussions regarding the chosen patient situation. These results coincide with a review highlighting the importance of helping students to understand and enhance the APN role by using advanced knowledge and research in their professional development [7]. Furthermore, our study found that the students felt more confident in their competence regarding ethical issues. In general, nurses’ reflections on ethics lead to greater self-confidence by clarifying their role and helping them find solutions to improve their clinical practice [24]. Moreover, competence in systematic reflection is necessary when working in clinical practice environments [25]. Reflection in groups has a particularly important role in developing one’s professional practice, and reflection-beyond-action can help students understand clinical situations by using a story in the context of a past experience. Reflection on clinical issues is also important in life-long learning for the further development of professional competence [18]. Participating in VRG meetings and reflecting on clinical issues improved the APN students’ ability to achieve a deeper understanding of complex patient situations.

The results show that the discussions in the VRG meetings stimulated the APN students to develop their critical thinking skills, as they received feedback from other group members. The APN students need to be trained in critical thinking because it allows them to actively participate in their learning process in developing competence as an APN [7]. Critical thinking is the process by which the students form a reflective judgment about what to believe or what to do in a given context [1]. Critical thinking is defined by the American Association of Colleges of Nursing as all or part of the process of questioning, analysis, synthesis, interpretation, inference, inductive and deductive reasoning, intuition, application, and creativity, which leads to clinical judgments and safe clinical decisions [4]. Another study found that the greatest development of competence was in relation to direct clinical practice. Although the students entered the program with different levels of competence, this was largely equalized during their education in the master’s program [26]. The data from the focus group in our study showed that the learning outcomes from the VRGs strengthened the students’ critical thinking skills in the process of developing their competence, in particular by taking increased responsibility for leading professional discussions.

The discussions in the VRGs promoted the strengthening of the collaboration between education and clinical practice. Implementation of the reflection groups as VRGs facilitated participation of the clinical supervisors together with the responsible student, meeting the group of students from other placements in different hospitals and the educator on the web in the meeting. The clinical supervisor who participated contributed to the updated clinical experiences in the professional discussion, which the students experienced as having a positive influence on their process of developing competence as an APN. The clinical supervisor contributed to the discussion with practical experiences to help students better understand the challenges APNs face in complex patient situations with a strong medical focus. APN students also need to learn medical subjects while maintaining a holistic approach that includes providing patient-centered care, which is the foundation of nursing [7]. Developing clinical decision-making is another important aspect of clinical competence [26,27]. APNs are experts in nursing who have extended their medical knowledge while retaining their unique nursing character [7]. In the process of helping students develop their professional APN role, the clinical supervisor acts as a role model in connecting medical topics to the assessment and management of the clinical situation in the chosen case.

The VRGs had implications for clinical practice as the supervisors, together with the students, continued their professional discussions after the meetings. The students reported in the focus group that the supervisors followed up in clinical practice on the issues from the discussions and the challenges identified in the VRG and together they continued to further reflect on and beyond the situations in the clinical unit [17,18]. During their reflections on the chosen case, the students gained a deeper understanding of the situation by developing increased situational awareness, as supported by the clinical supervisor. An important learning outcome in the master’s program is to achieve increased situational awareness in complex patient situations [14]. The students had useful experiences cooperating with the supervisor in linking research to the chosen situation from practice when preparing for the VRG meetings. Together, they brought the new knowledge back to the clinical practice for further assessment and implementation, which gave the APN students useful and relevant experiences in preparation for the APN role. Developing evidence-based practice through the critical assessment of research is part of an APN’s core competence [13]. The APN students are, according to the results from a Swedish study, engaged in the process of gaining a more advanced identity for the APN role, which is based on both practical and theoretical knowledge [27]. Developing the role as an APN includes advanced knowledge in critical thinking, clinical decision-making, and comprehensive assessment [7,8]. In professional discussions in the VRGs, the APN students took on increased responsibility in collaboration with the clinical supervisor in preparing the case. Together, they followed up on the issues from their reflections by continuing professional discussions in clinical practice. The students’ experiences from their reflections in the VRGs increased their competence in preparing them for the APN role.

The study has implications for practice, as the results provide useful information that supports the use of the VRG, which increases the possibility of clinical supervisors joining the meetings and supports the students in developing their new APN role. The digital competence achieved throughout the COVID-19 lockdown will prove useful when continuing to conduct VRGs after the pandemic, and further use of VRGs will continue to strengthen the collaboration between health care institutions that offer clinical practice and educational institutions that are responsible for ensuring the quality of nursing education.

### Limitations

This study has some limitations, as only 1 focus group interview was conducted. The interview was completed after the last period of the students’ clinical placement during an APN master’s program, with 6 students enrolled in this course participating in the focus group interview. However, although it was only 1 interview, it generated rich data that provided sufficient information power [28]. The 6 students had experience with different clinical placements in different hospitals and were divided into different groups for the VRGs. Only students were selected as respondents for this study. Including clinical supervisors in this study could have provided useful information from their point of view.

### Conclusions

Reflection groups were implemented as VRG meetings using web-based communication during the lockdown because of the COVID-19 pandemic. The rigorous guidelines for the VRGs ensured a solid structure for the meetings, and professional discussions stimulated APN students’ critical thinking and the development of competencies. The participants responsible for the VRG meetings experienced increased responsibility as they were in charge of organizing the meetings, chose a patient case, and found research related to the patient case. The students in the respondent role reported their development of critical assessment in the clinical and research contexts, as they were responsible for giving feedback on the chosen patient case and the research article. This study found that participating in VRG meetings challenged APN students to reflect on the competence they had developed in their master’s education. They experienced achieving increased awareness of the APN role in complex clinical patient situations during clinical placement. As the VRG meetings were web-based, the clinical supervisor could participate with the educators and students from placements in different hospitals. The clinical supervisors contributed by sharing their actual experiences, which improved the professional discussions. The APN students and clinical supervisors continued the discussions in the clinical settings afterward, which strengthened the collaboration between education in the master’s program and clinical practice.

## Abbreviations

APN: advanced practice nurse
VRG: virtual reflection group
